# Supplementary material for: Decoding murine cytomegalovirus
Source: PLoS Pathog. 2023 May 12;19(5):e1010992. doi: 10.1371/journal.ppat.1010992 (PMC10208470; doi:10.1371/journal.ppat.1010992)
Supplement: S1 File — (DOCX) [file ppat.1010992.s001.docx]

**Datasets**

The following datasets were obtained for the analysis of all high-throughput sequencing data as described in **Fig 1**. All infections were conducted in NIH-3T3 cell lines with centrifugal enhancement (800g for 30 min).

(1) Total RNA-seq data from mock, 1, 2, 6, 12, 24 and 48 h.p.i. (n=2)

(2) 4sU-seq data obtained from mock, 1, 2, 6, 12, 24 and 48 h.p.i. (n=2)

The above data sets involved sequencing of RNA collected at the above time points for both infected (MOI:10) and mock infected samples. For 4sU-seq, 500µM 4sU was used to label nascent RNA for 60 minutes prior to lysis. RNA obtained was purified and analysed as described (*1*).

The data of (1) and (2) were deposited as [GSE212287](https://www.ncbi.nlm.nih.gov/geo/query/acc.cgi?acc=GSE212287).

(3) cRNA-seq samples from mock, 2, 6, 12, 24, 48 h.p.i. (n=2)

cRNA-seq is a modified total RNA sequencing protocol based on the circularization of RNA fragments. This circularization step leads to the enrichment of reads at 5’ ends of transcripts, i.e., at transcription start sites. RNA isolated from the above-mentioned time-points for both infected (MOI:10) and mock-infected samples was harvested and subject to the cRNA-seq protocol as described (*1*). The respective data was deposited as [GSE212285](https://www.ncbi.nlm.nih.gov/geo/query/acc.cgi?acc=GSE212285)

4) dRNA-SLAM-seq samples from mock,1,2,4,6,8,12,24,36,48 and 72 h.p.i. (n=2)

dRNA-SLAM-seq for transcription start site profiling involved labelling of nascent RNA with 400µM 4sU 1 hour prior to lysis. RNA extracted via Trizol was subject to SLAM-seq alkylation using iodoacetamide (IAA) and re-purified to prepare libraries to generate this dataset. The respective data was deposited as [GSE212286](https://www.ncbi.nlm.nih.gov/geo/query/acc.cgi?acc=GSE212286)

5) Ribosome profiling from mock, 2, 6, 12, 24, 48 hpi (n=4) along with translation start site profiling using Harringtonine (2, 6, 24 hpi, n=2) and Lactimidomycin (24 hpi, n=1) pre-treatment.

Samples were subjected to RNase1 treatment followed by ribosome isolation and recovery of ribosome-protected RNA fragments and library preparation. Harringtonine and Lactimidomycin were used to arrest ribosomes at translation start sites. The respective data was deposited as [GSE212288](https://www.ncbi.nlm.nih.gov/geo/query/acc.cgi?acc=GSE212288).
