# Supplementary figures and images for: Decoding murine cytomegalovirus
Source: PLoS Pathog. 2023 May 12;19(5):e1010992. doi: 10.1371/journal.ppat.1010992 (PMC10208470; doi:10.1371/journal.ppat.1010992)

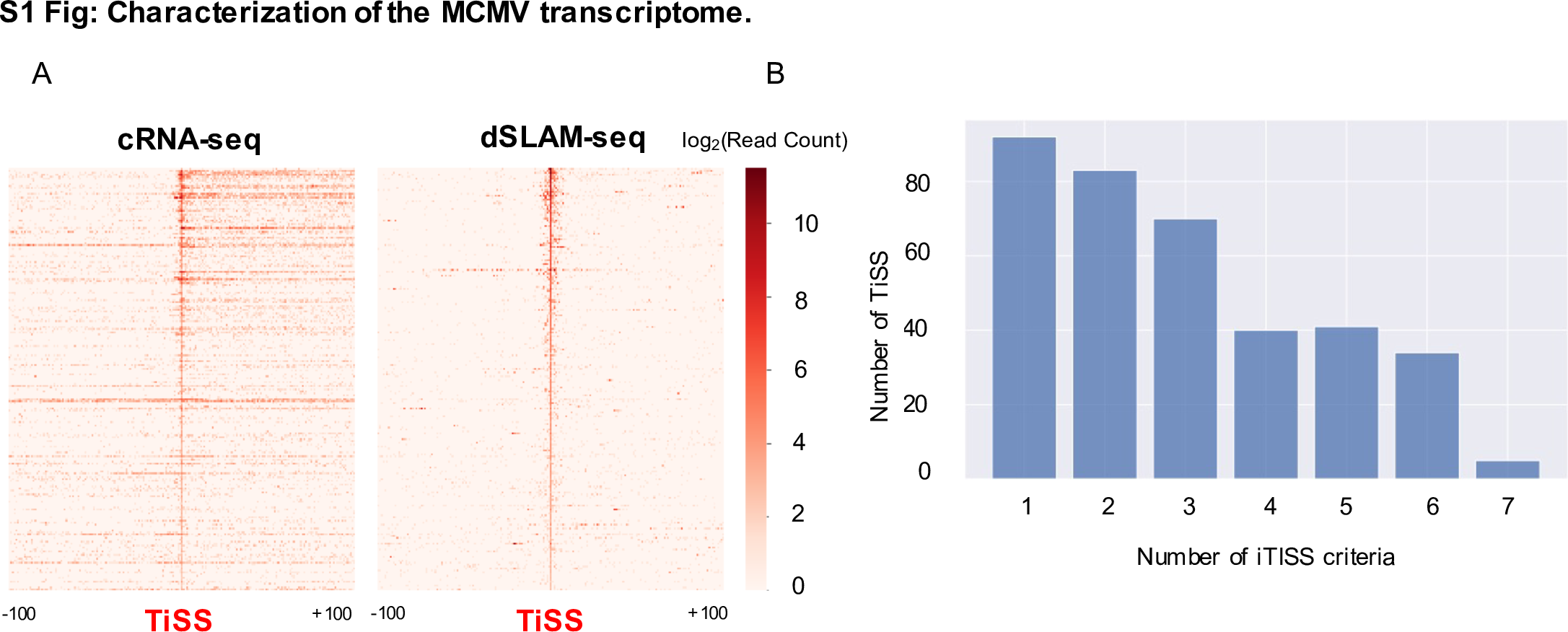

Supplement: S1 Fig — A. Heat maps comparing read enrichment at transcription start sites (TiSS) in the cRNA-seq and dSLAM-seq data. The x axis represents distance from TiSS (+/-100 bp) for 222 TiSS displayed along the y-axis detected by both dSLAM-seq and cRNA-seq. Colors represent maximal read count in log2 scale across all samples in cRNA-seq and dSLAM-seq, respectively. B. Histogram depiction of the number of MCMV TiSS satisfying the indicated number of criteria of the iTiSS algorithm. A detailed description of the employed criteria is included in methods. Scores for all TiSS assigned by iTiSS are shown in S4 Table. (TIF) [file ppat.1010992.s014.tif]

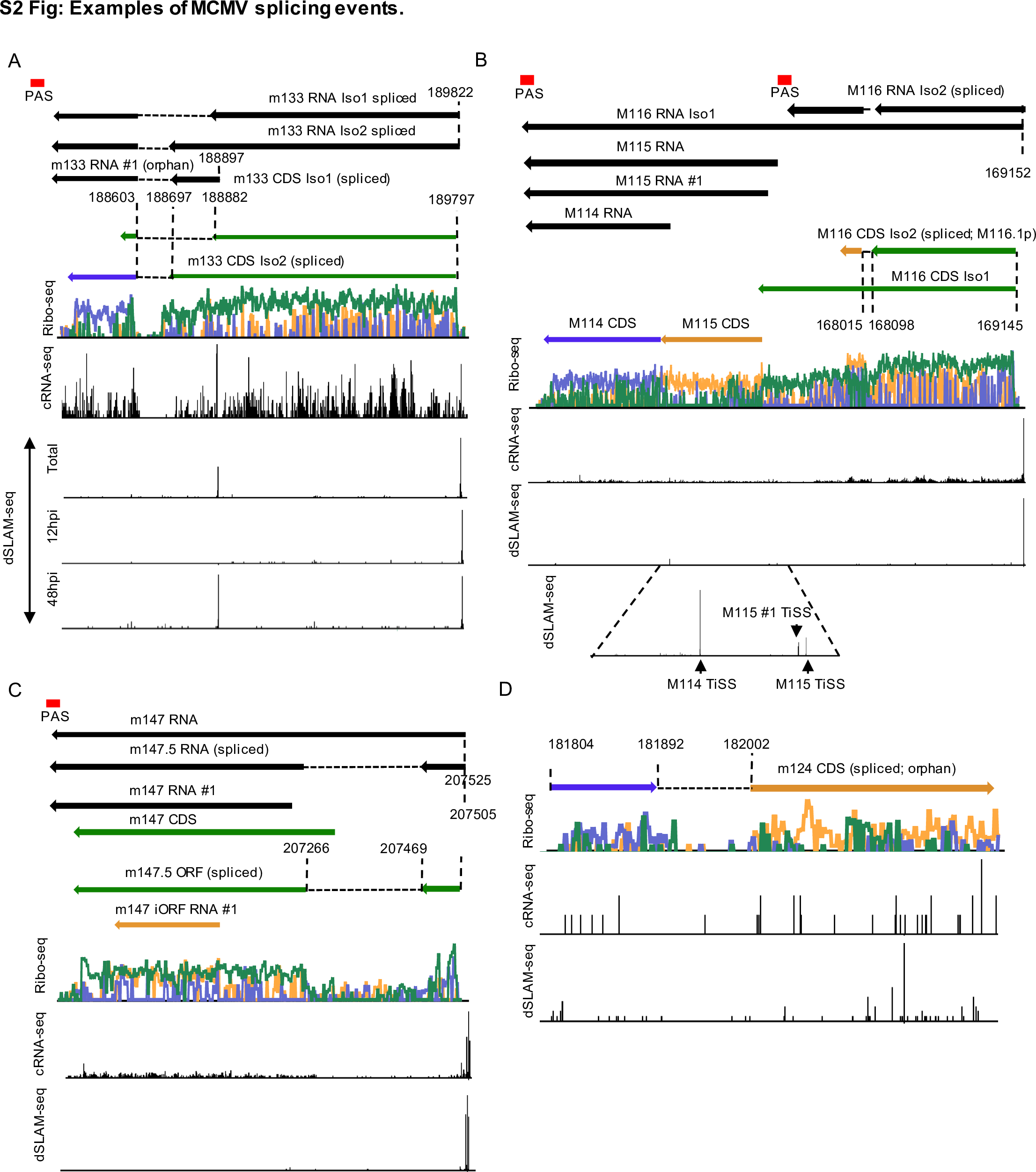

Supplement: S2 Fig — Each schematic depicts viral gene expression and splicing in a given locus. Aggregated reads of Ribo-seq, cRNA-seq and dSLAM-seq data across all time points of infection are shown. Ribo-seq data are indicated in logarithmic scale, cRNA-seq and dSLAM-seq data in linear scale. The arrows at the top depict the annotated transcripts (black), poly(A) sites (PAS; in red) and ORFs (colored depending on the translated frame (yellow, purple and green)). The bold dotted line represents introns detected by 4sU-seq. A. In the m133 locus, splicing of two introns leads to the expression of both a known (Iso1) and a novel spliced ORF (Iso2), the latter is expressed by an alternative donor site, as predicted by Rawlinson et al. [22]. The m133 RNA #1 (orphan) transcript did not bear any evidence for downstream translational start sites and was annotated as a spliced transcript with delayed late kinetics, spliced similarly to m133 RNA Iso2 as evident from the overall read accumulation in cRNA-seq and dSLAM-seq (48 hpi) B. In the M116 locus, splicing explained a truncated M116 CDS Iso2 (M116.1p) revealed by ribosome profiling, whose transcript may terminate at an earlier poly(A) site (PAS). A second PAS downstream serves for the transcript encoding the unspliced M116 CDS. Here, transcription continues past the first PAS resulting in M116 RNA Iso1 which overlaps with the M115 and M114 transcripts whose expression levels correspond to their respective ORFs and were hence annotated. High levels of gene expression across the M116 locus resulted in a number of putative TiSS depicted in the dSLAM-seq with no evidence of downstream translation, uniform cRNA-seq read distribution and overall lower gene expression as compared to the canonical TiSS. Hence, they were attributed to experimental noise and not annotated. C. In the m147.5 locus, splicing leads to the expression of a previously validated spliced ORF. D. In the m124 locus, splicing necessitates correction of the previously annotated m [file ppat.1010992.s015.tif]

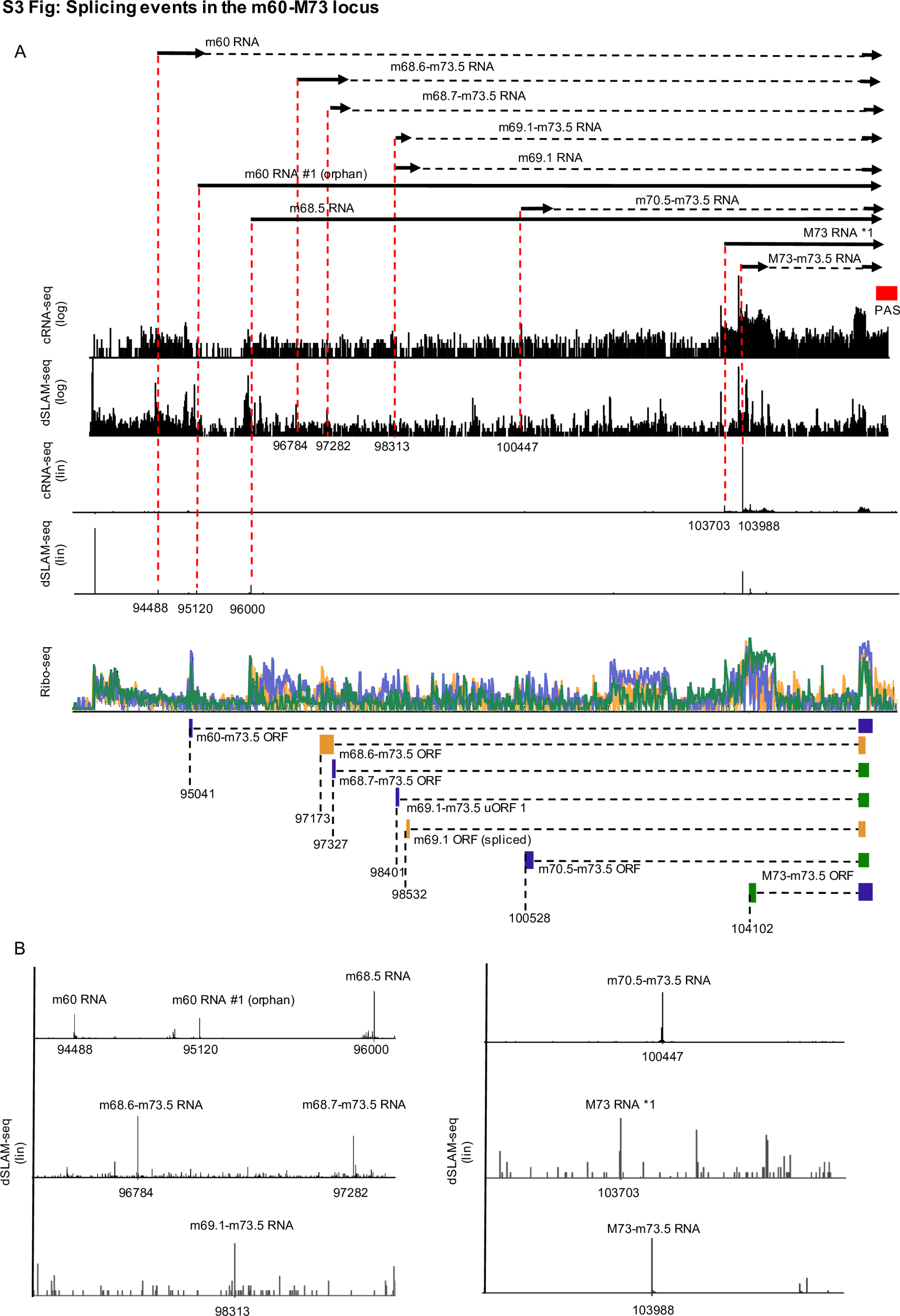

Supplement: S3 Fig — Graphs represent TiSS profiling data (black) from cRNA-seq and dSLAM-seq as well as ORFs called by Ribo-Seq (different colors represent different frames of translation). Aggregated reads of Ribo-seq, cRNA-seq and dSLAM-seq data across all time points of infection are shown. Ribo-seq data are indicated in logarithmic scale, cRNA-seq and dSLAM-seq data in both linear and logarithmic scale. A. Spliced ORFs are depicted by exons connected with a dotted line representing introns at the bottom. Multiple splicing events were observed in the m60-73.5 locus, of which the m60 RNA and M73-m73.5 spliced transcripts have already been validated previously (see S2 Table). Of note, translation occurs in different fames upstream of splicing thereby explaining translation in different frames in the common downstream exon. For a given frame of translation at the second exon, the expression levels correlated well with the respective upstream exons. B. Shown is a zoomed schematic view of the dSLAM-seq data portraying every TiSS depicted in panel A in linear scale. Coordinates of the start codons/exon start sites and TiSS for all spliced events are indicated. (TIF) [file ppat.1010992.s016.tif]

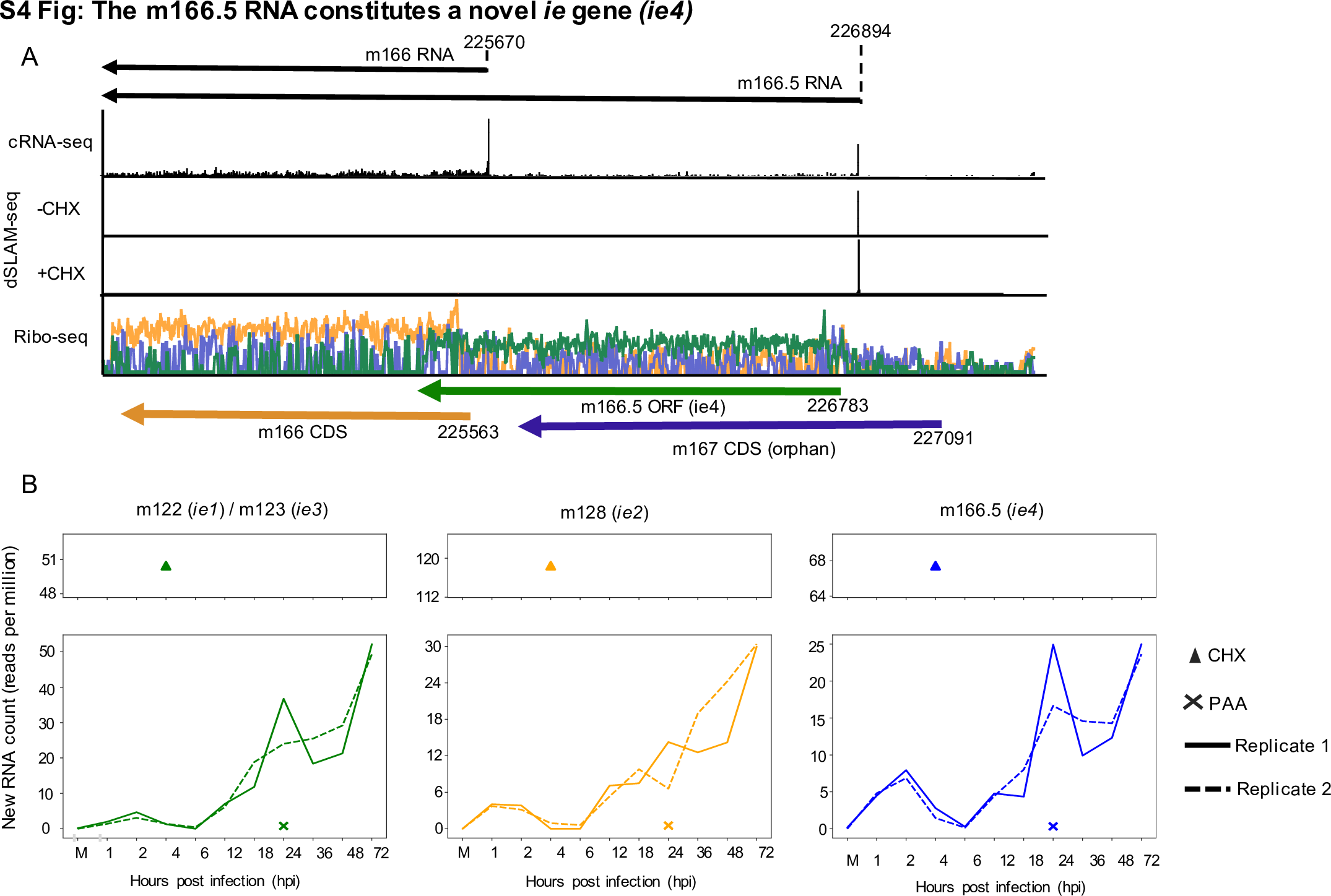

Supplement: S4 Fig — A. Cycloheximide (CHX) treatment combined with dSLAM-seq identified a so far unknown viral immediate early transcript in the m166.5 locus. Aggregated reads of Ribo-seq, cRNA-seq and dSLAM-seq data across all time points of infection in the m166-m167 locus are shown. Ribo-seq data are depicted in log scale, cRNA-seq and dSLAM-seq data (-/+CHX; 4 hpi) in linear scale. The m166.5 immediate-early transcript (termed ie4) and its corresponding m166.5 ORF overlap with the m167 CDS (orphan) and partially overlap with the N-terminal part of the m166 CDS. Unlike the m166 RNA, m166.5 RNA is expressed despite CHX pre-treatment implying immediate-early gene kinetics. B. Line graphs representing gene expression (new RNA levels) of the three ie genes (four when counting ie1 and ie3 as separate genes despite their use of the same TiSS) over time for two replicates per gene. New RNA expression levels of phosphonoacetic acid (PAA; 24 hpi)- or CHX (4 hpi)-treated samples (n = 1) are indicated as a star/triangle respectively for a given gene. Increase in new RNA levels for a given gene under CHX treatment defines them as immediate-early genes (TR0). Coordinates of the start codons and TiSS of all indicated gene products are displayed. (TIF) [file ppat.1010992.s017.tif]

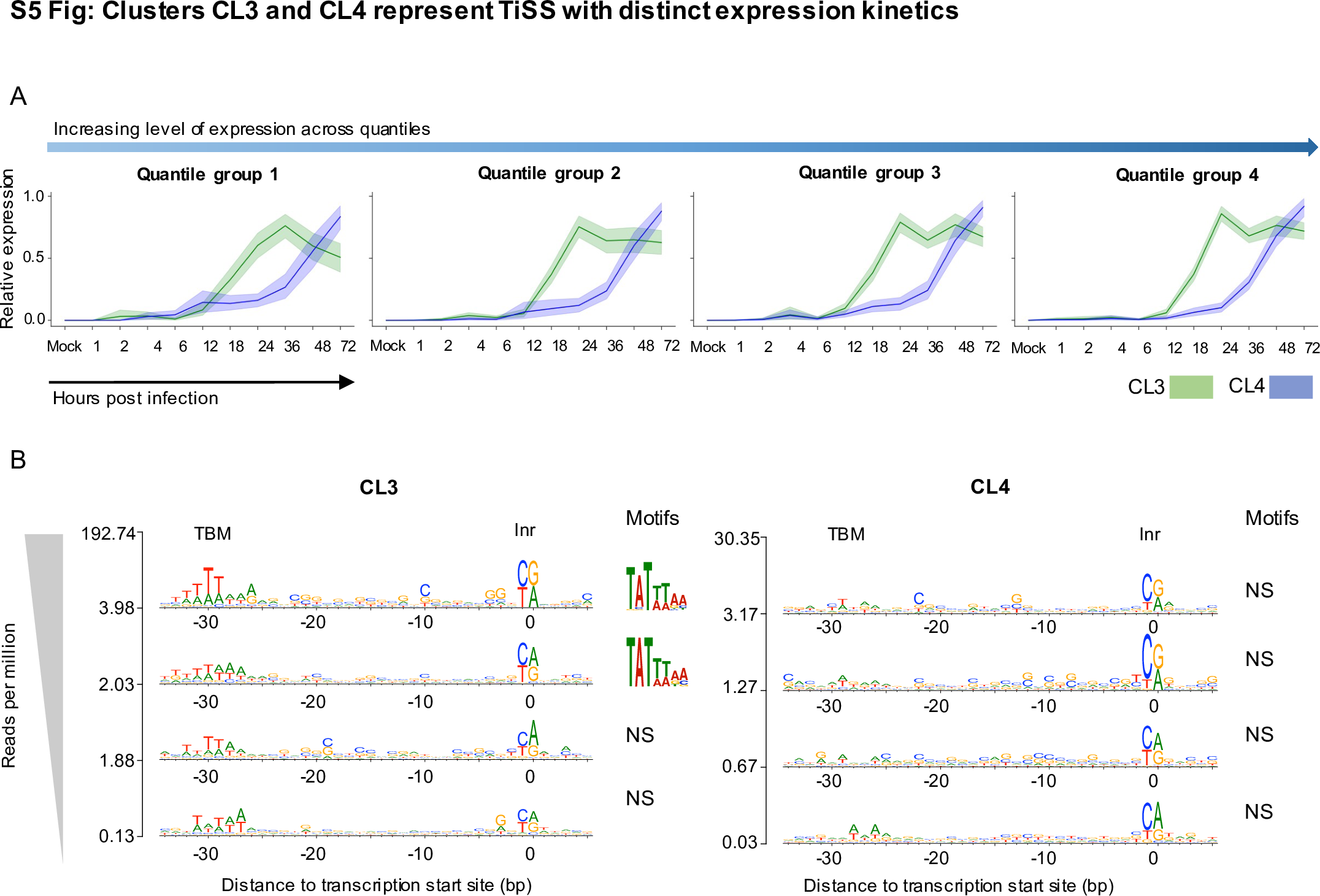

Supplement: S5 Fig — A. Quantile groups segregated according to levels of expression for CL3 and CL4 transcripts on the basis of new RNA for the respective viral TiSS obtained from dSLAM-seq data. The x-axis displays hours post infection. Relative expression is shown on the y-axis. CL3 and CL4 transcripts are indicated by green and blue lines, respectively. B. Motif analysis (MEME) for all four quantiles for CL3 and CL4 transcripts. NS: Not significant. (TIF) [file ppat.1010992.s018.tif]

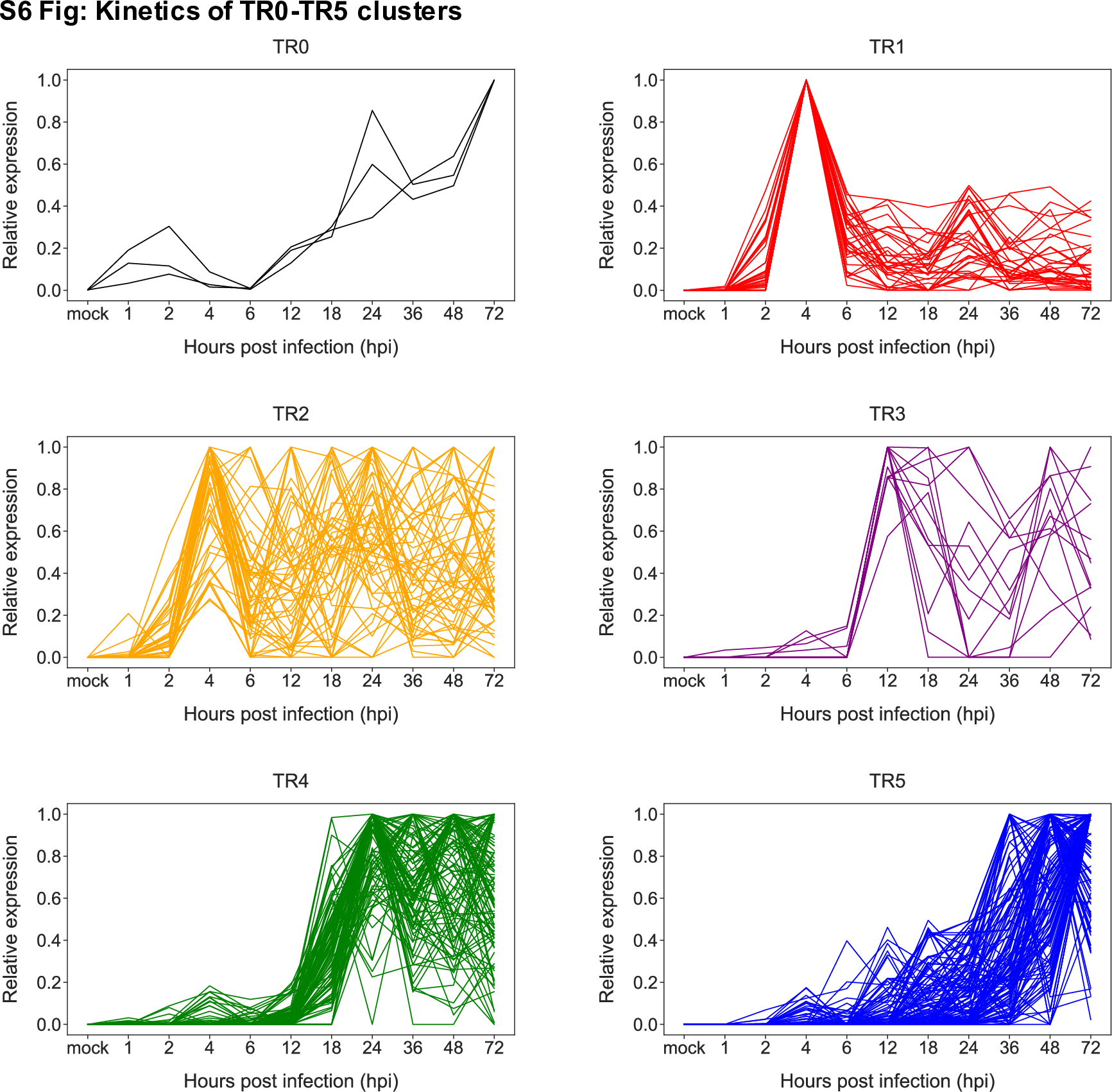

Supplement: S6 Fig — Line graphs representing all TiSS within the six TR clusters (TR0-5). The y-axis represents relative new RNA levels across the infection time-course (x-axis). (TIF) [file ppat.1010992.s019.tif]

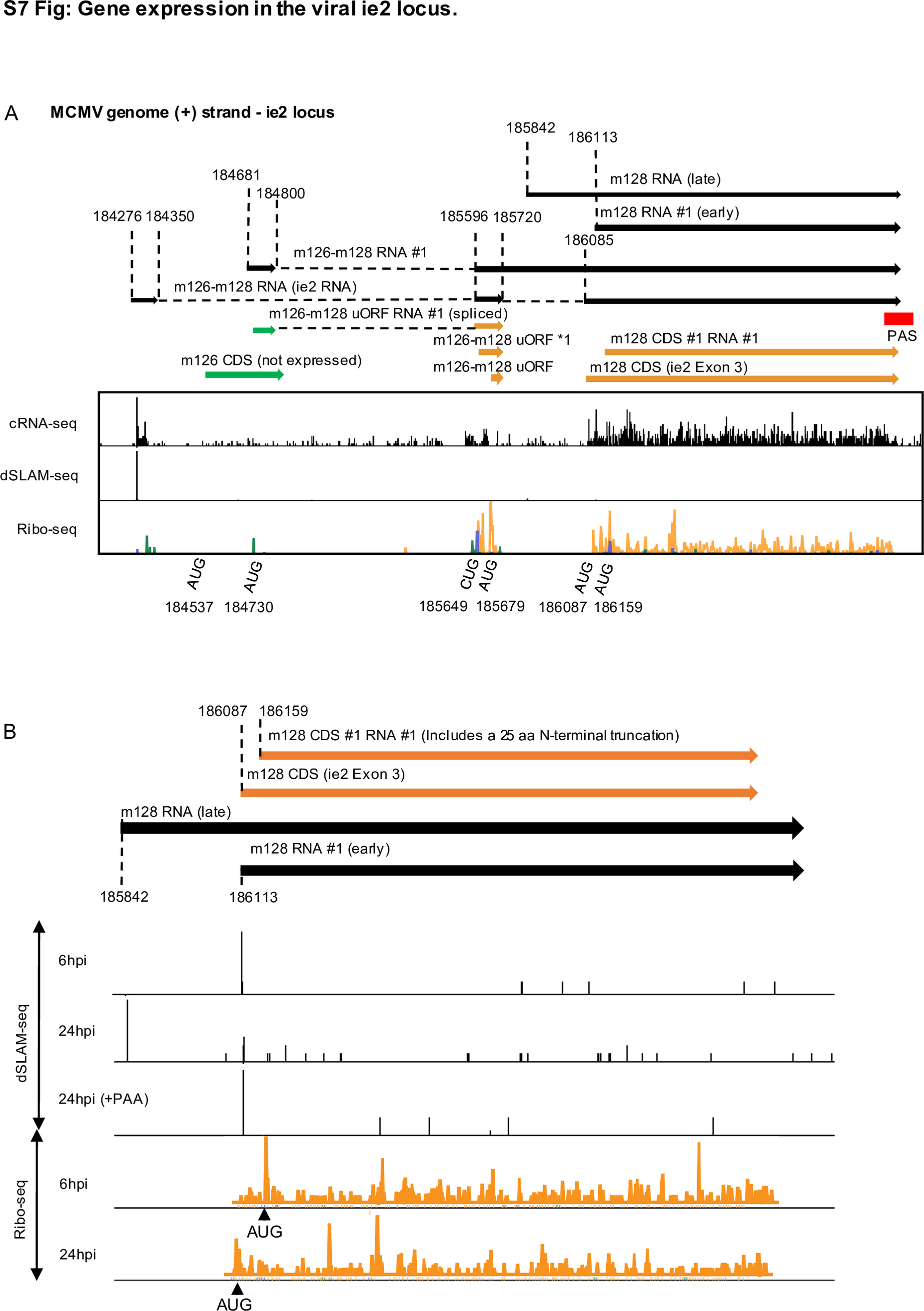

Supplement: S7 Fig — A. Schematic of the ie2 locus. The canonical TiSS is represented by the dominant spliced ie2 transcript (m126-m128 RNA) comprising 2 introns and only one ie2 coding exon initiating at the first AUG (186087) shown i.e., m128 CDS (ie2 Exon 3). A second AUG represents a truncated isoform (m128 CDS #1 RNA #1). Aggregated reads of Ribo-seq, cRNA-seq and dSLAM-seq data across all time points of infection are shown. Ribo-seq data are indicated in log scale, cRNA-seq and dSLAM-seq data in linear scale. B. Graphs represent TiSS profiling data (black) from dSLAM-seq including kinetics for 6 and 24 hpi and ORFs called by Ribo-seq (Colored) for the same time points for a given replicate. The arrows above depict manual annotations of transcripts and ORFs. Alternative transcription initiation at the ie2 (m128) locus led to the expression of an N-terminally truncated ORF expressed from an early TiSS (m128 RNA #1) whose expression was not influenced by PAA treatment. cRNA-seq and dSLAM-seq data are represented in linear scale, Ribo-seq in logarithmic scale. Coordinates of all TiSS, start codons and spliced junctions in the m126-m128 locus are displayed. (TIF) [file ppat.1010992.s020.tif]

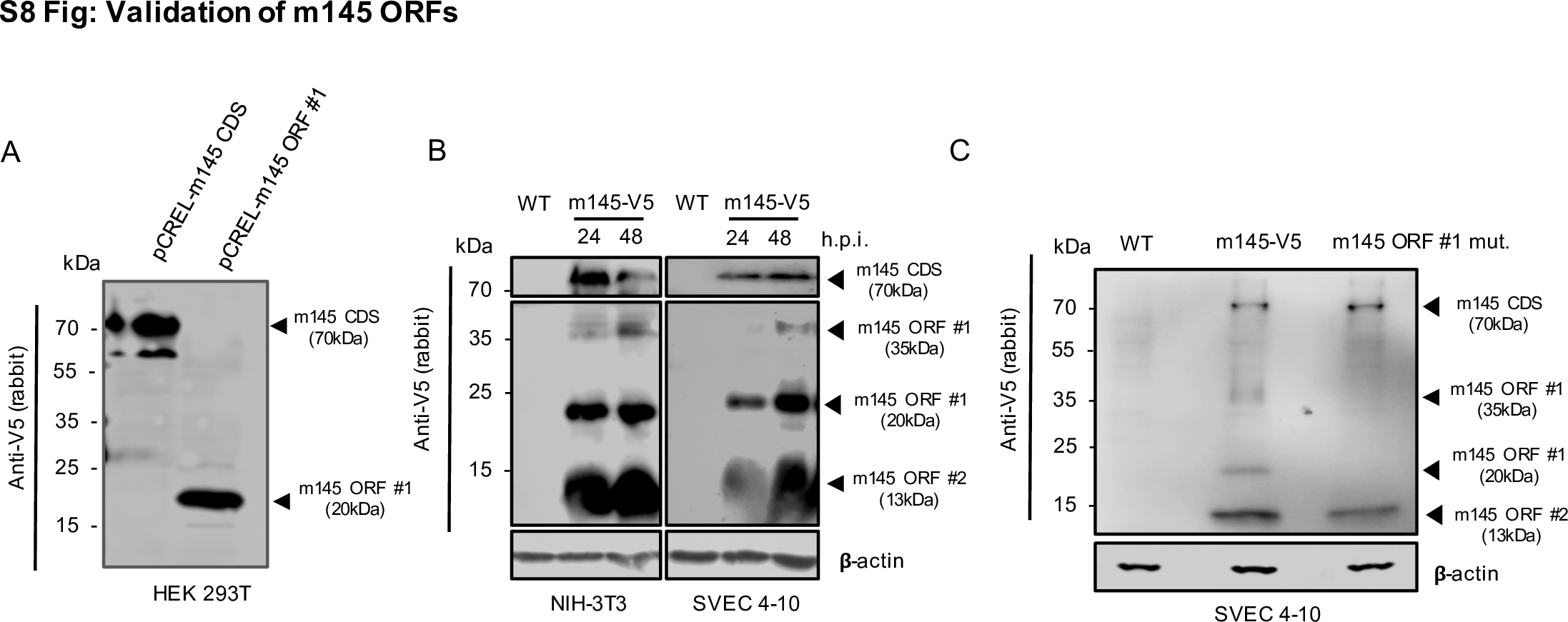

Supplement: S8 Fig — Both the m145 CDS and m145 ORF #1 were cloned into expression plasmids (pCREL-IRES-Neon) with their expression driven by a CMV promoter. A. Expression of the two viral ORFs was validated via transfection of the respective plasmids into HEK293T cells. Western blots were performed at 48 h post transfection. Both the 55 kDa (non-glycosylated) and 70 kDa isoforms of m145 CDS were detected whereas only a single 20 kDa isoform of m145 ORF #1 was detected. B. The m145-V5 virus described in Fig 7B was used to infect both NIH-3T3 and SVEC 4–10 cells at an MOI of 1 for the respective time points to validate the m145 gene products, whose expression was similar in both cell lines. C. A start codon mutant of m145 ORF #1 (△m145 ORF #1 mut) was utilized to infect SVEC 4–10 cells for 48 h. Western blot analysis revealed expression of m145 ORF #2 to remain unaffected. WT indicates wild-type MCMV. β-actin was used as a housekeeping control. Images are a single representative of 2 biological replicates (n = 2) for each experiment. Additional gene products (35 kDa and 13 kDa) of m145 RNA #1 were not detected in the expression plasmid system in S7A. Absence of the 13 kDa isoform (m145 ORF #2) is likely to be due to the optimized Kozak sequence of the employed expression vector, which prevents ribosomes from bypassing the main AUG start codon. The reason for the absence of the 35 kDa isoform remains unclear. We hypothesize that this may be due to differences in the employed cell system or requirements for other viral gene products. (TIF) [file ppat.1010992.s021.tif]
